# An effective hybrid of hill climbing and genetic algorithm for 2D triangular protein structure prediction

**DOI:** 10.1186/1477-5956-9-S1-S19

**Published:** 2011-10-14

**Authors:** Shih-Chieh Su, Cheng-Jian Lin, Chuan-Kang Ting

**Affiliations:** 1 Department of Computer Science and Information Engineering, National Chung Cheng University, Chiayi 62102, Taiwan, R.O.C; 2 Department of Computer Science and Information Engineering, National Chin-Yi University of Technology, Taichung 41101, Taiwan, R.O.C

## Abstract

**Background:**

Proteins play fundamental and crucial roles in nearly all biological processes, such as, enzymatic catalysis, signaling transduction, DNA and RNA synthesis, and embryonic development. It has been a long-standing goal in molecular biology to predict the tertiary structure of a protein from its primary amino acid sequence. From visual comparison, it was found that a 2D triangular lattice model can give a better structure modeling and prediction for proteins with short primary amino acid sequences.

**Methods:**

This paper proposes a hybrid of hill-climbing and genetic algorithm (HHGA) based on elite-based reproduction strategy for protein structure prediction on the 2D triangular lattice.

**Results:**

The simulation results show that the proposed HHGA can successfully deal with the protein structure prediction problems. Specifically, HHGA significantly outperforms conventional genetic algorithms and is comparable to the state-of-the-art method in terms of free energy.

**Conclusions:**

Thanks to the enhancement of local search on the global search, the proposed HHGA achieves promising results on the 2D triangular protein structure prediction problem. The satisfactory simulation results demonstrate the effectiveness of the proposed HHGA and the utility of the 2D triangular lattice model for protein structure prediction.

## Introduction

Since the presence of HP lattice model [1], heuristic search algorithms for a variety of lattice models have been proposed and proven useful to explore the relationship between the primary amino acid sequence and its native folding structure, particularly in the protein folding problem (PFP) and the protein structure prediction (PSP). The main purpose of the HP lattice model is to understand the physicochemical principle of protein folding during the modeling process of searching for the lowest free-energy conformation of a protein.

Despite the difference in modeling accuracy, both high-resolution and low-resolution models can contribute to an understanding of the protein structure obtained from experiments, such as NMR and crystallography. Moreover, they have various applications in protein modification, protein-ligand and protein-protein interactions [2]. Table 1 summarizes the relationship between modeling accuracy and the related applications.

**Table 1:** The relationship between modeling accuracy and the related application.

Accuracy	Application
<30%	Refining NMR structures Finding binding/active sites by 3D motif searching Annotating function by fold assignment

30%-60%	Molecular replacement in crystallography Supporting site-directed mutagenesis

>60%	Comparable to medium-resolution NMR, low-resolution crystallography Docking of small ligands, proteins

This table illustrates the accuracy of protein modeling and its related application [2].

**Figure 1:**
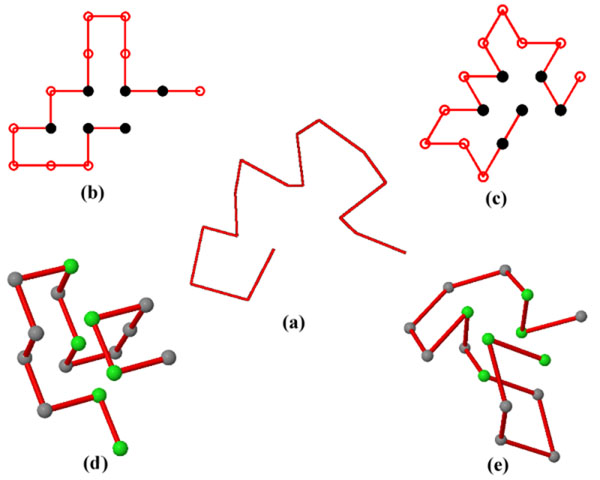
**Four different types of lattice model for visual comparison taking the protein with the PDB id: 1A0Ma as the example**. Visual comparison for PDB id: 1A0Ma. (a) Real protein structure; (b) and (c) are 2D square and triangular lattice model simulation results. Black-filled dots indicate Hydrophobic amino acids and white dots denote hydrophilic amino acid. (d) and (e) are 3D square and face-centered cubic (FCC) lattice model simulation results from CPSP-tools [3]. In (d) and (e), green balls indicate hydrophobic amino acids while the gray balls indicate the hydrophilic amino acids.

To improve the modeling accuracy, several lattice models have been developed and proposed. The present study compares four popular lattice models in terms of visual comparison, including 2D square and triangular lattice models, 3D cubic lattice model and face-centered cubic (FCC). The protein structures obtained from the four modeling types were compared with reported 'real' biological protein structures. As Figure 1 shows, the 2D triangular lattice model can give a better structure modeling and prediction for proteins with short primary amino acid sequences.

In solving this prediction problem, Hart and Istrail [4] first gave a 1/4 (25%) approximation for the problem of the 2D square lattice and a 3/8 (38%) approximation for the problem of the 3D cubic lattice. Agarwala et al. [5] gave a 6/11 (54%) approximation for the problem, which is consistent with our experimental results.

Many researchers have favored and focused research on the square lattice model because it has many associated benchmarks, large amount of data accumulated over the years, and the availability of comparison with different strategies and modeling methods. By contrast, little work has been done on the 2D triangular lattice model. In this paper, we proposed a genetic algorithm with elite-based reproduction strategy (ERS-GA). Based on ERS-GA, this study further develops a hybrid of hill-climbing and genetic algorithm (HHGA) for protein structure prediction on the 2D triangular lattice. Experimental results were conducted to validate the effectiveness of this method.

The remainder of this paper is structured as follows: Section II gives the preliminaries and the definition of the protein structure prediction problem in the HP 2D triangular lattice model. Section III describes the methodology used in the study. The comparison of results is presented and discussed in Section IV followed by the conclusion in Section V.

## Preliminaries

Proteins play fundamental and crucial roles in nearly all biological processes, such as, enzymatic catalysis, signaling transduction, DNA and RNA synthesis, and embryonic development. It has been a long-standing goal of molecular biology to predict the tertiary structure of a protein from its primary amino acid sequence [6,7]. This paper emphasizes research on *ab initio* modeling, among which the 2D HP triangular lattice model is thought to be the best two-dimensional model in protein structure prediction at present.

### HP lattice model

**Figure 2:**
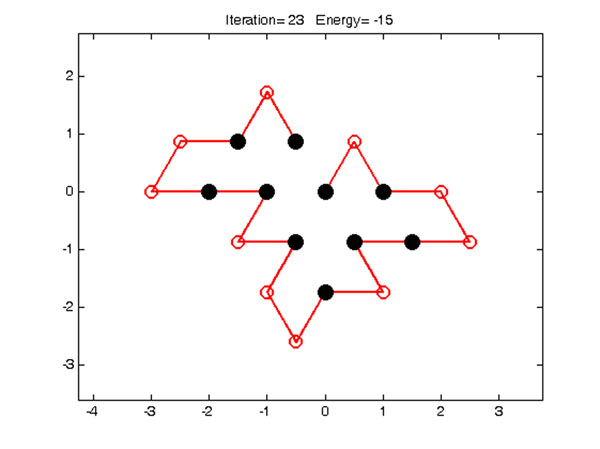
**An optimal conformation in a 2D triangular lattice model**. An optimal conformation for the sequence (HP)^2^PH(HP)^2^(PH)^2^HP(PH)^2^ in a 2D triangular lattice model. The black filled dots denote the hydrophobic amino acid and the red open circles denote the hydrophilic amino acids. The H-H contacts (free energy) in the conformation are assigned the energy value of -1. The free energy is defined as a minimum value; the maximum number of H-H contact is given in the case of two-dimensional models, Figure 2 illustrates a protein structure with 15 H-H contacts (energy= -15).

The HP lattice model [1] is the most frequently used model, which is based on the observation that the hydrophobic interaction between amino acid residues is the driving force for protein folding and for development of native state in proteins [8]. In this model, each amino acid is classified based on its hydrophobicity as an H (hydrophobic or non-polar) or a P (hydrophilic or polar). The HP lattice model allows HP protein sequences to be configured as self-avoiding walks (SAW) on the lattice path favoring an energy free state according to HH interaction. The energy of a given conformation is defined as the number of topological neighboring (TN) contacts between H's that are not adjacent in the sequence. Figure 2 shows an example for the 2D triangular lattice model.

### Calculation of free energy

The free energy of a protein can be calculated by the following formulae [9]:(1)(2)

where the parameter(3)

Protein folding can then be transformed into an optimization problem for the conformation with minimal free energy. Formally, given an HP sequence *s* = *s*_1_*s*_2_…*s*_n_, find a conformation of *s* with minimum energy. That is, the problem is to find c* ∈ *C*(*s*) such that *E*(c*) = min{*E*(*c*)|*c* ∈ *C*(*s*)}, where *C*(*s*) is the set of all valid conformations for *s *[*10*].

### Triangular lattice model

**Figure 3:**
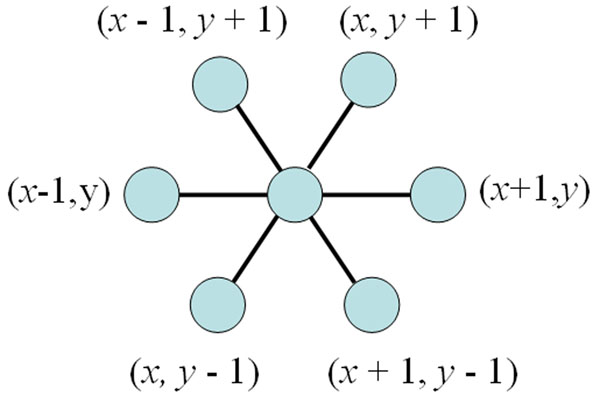
The 2D triangular lattice model neighbors of vertex (x, y).

A significant drawback of the cubic lattice [5] is that, if two residues are at any even distance in the primary sequence, they cannot be in topological contact with one another when the protein is embedded in this lattice. In other words, on the square lattice, two amino acids in contact in any folding must be at odd distance away in the protein sequence [5]. To address this issue, Joel et al. [11] introduced the 2D triangular lattice model. As Figure 3 shows, each lattice point has six neighbors in the two-dimensional triangular lattice. Since each residue has two covalent neighbors, except the first and the last residues, a residue at a lattice point can be in topological contact with at most four other residues. Thus, each residue is involved in up to four H-H contacts [11].

With the unit vectors obtained from the triangular lattice, it is much easier to model protein conformation on a two-dimensional triangular lattice without exhibiting the parity problem [5]. However, the lattice model of protein conformation as a self-avoiding walk is NP-complete [12]. To solve this problem, some heuristic search algorithms [13-18] have been developed for various lattice models. Backofen and Will [21] utilized advanced techniques such as constraint programming to calculate all optimal side-chain structures of a given sequence, and proved their optimality [3]. Further, Böckenhauer et al. [15] extended the library by implementing the 2D triangular lattice and the pull move set for triangular lattice models.

In this paper, we developed an effective hybrid of local search and genetic algorithm (GA) to resolve this problem. The performance is examined and compared to the results in [15]. More details about the proposed algorithm are presented in the next section.

## Methods

**Figure 4:**
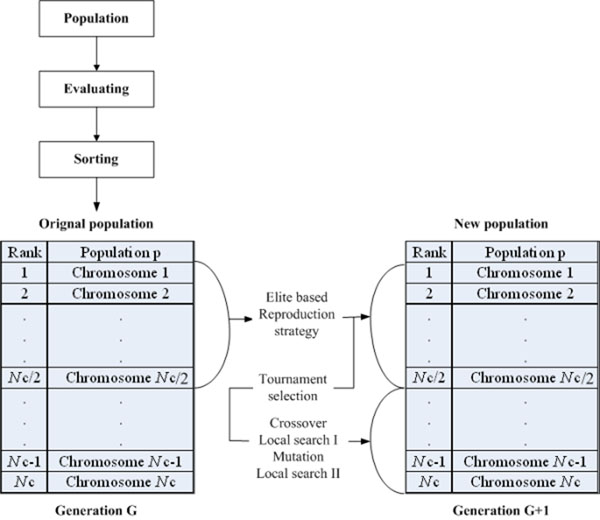
Flowchart of the elite-based reproduction strategy (ERS).

**Figure 5:**
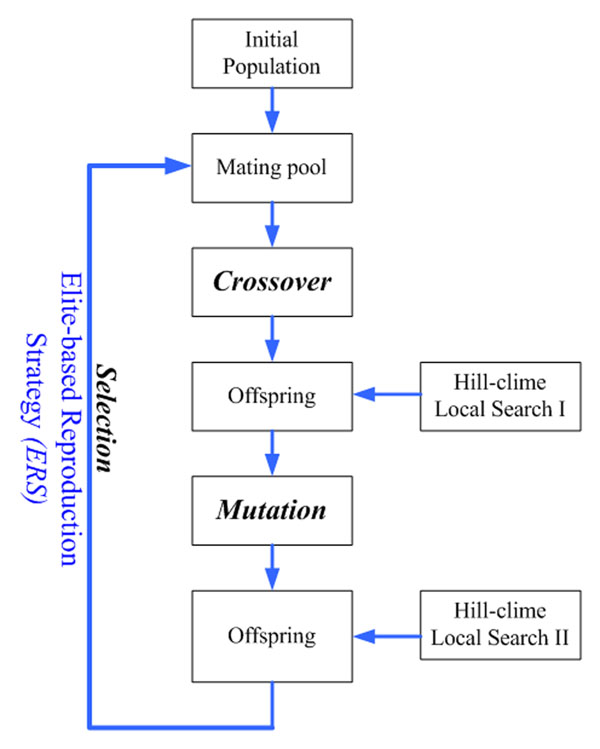
Flowchart of the hybrid of hill-climbing and genetic algorithms (HHGA)

This paper introduces the elite-based reproduction strategy to GA as the ERS-GA. Further, we propose a hybrid of hill-climbing and ERS-GA, called the HHGA, for protein structure prediction on the 2D triangular lattice. The proposed HHGA, in essence, is a combination of global search algorithm with local search operator. Restated, HHGA works within the framework of ERS-GA and adopts hill-climbing to enhance its exploitation capability. Figures 4 and 5 show the flow charts of the proposed ERS-GA and HHGA. The following subsections describe the operators of ERS-GA and HHGA.

### Initialization

For an input amino acid sequence of length *n*, a candidate conformation in the 2D triangular lattice [11,14] is encoded as a chromosome in the form of a string of length (*n* – 1) over symbols {*L*, *R*, *LU*, *LD*, *RU*, *RD*}, denoting the fold directions left, right, left-up, left-down, right-up and right-down, respectively. An initial population is generated randomly in the (n – 1) dimensional space within a predetermined range. In this paper, population size was set at 200 empirically.

Each chromosome in the population needs to be evaluated for its fitness. Here we directly use equation (2) of free energy as the fitness function. The goal for an optimization algorithm like HHGA is to minimize the fitness value, namely, free energy. The evaluated chromosomes are sorted according to their fitness values. This sorted population serves as the basis of subsequent reproduction process.

### Elite-Based Reproduction Strategy (ERS)

Reproduction is a process in which the information of candidate solutions are modified and copied, depending upon their fitness values. The reproduction in GA consists of selection, crossover, and mutation. For the ERS-GA and HHGA, this study adopts the elite-based reproduction strategy, which keeps the top half of the population to the next generation and generates offspring by performing crossover and mutation on the second half of the population [19]. In the experiments, this study uses two-point crossover with crossover ate 0.8 and uniform mutation with mutation rate 0.4.

### Local search

Two local search operators are proposed for the protein structure prediction problem. First, given the current solution, local search I chooses its neighbor residues, which are generated in a way similar to mutation operation: i.e., randomly changing its direction. Consequently, if the fitness value of a neighbor is better than the current solution, this neighbor residue will be accepted to replace the current one.

In local search II, the neighbor residues are generated in a way similar to crossover operation. That is, five neighbors are created by changing the direction of the second segment after the crossover point, where rotation angles are *60*°, *120*°, *180*°, *240*° and *300*°, respectively. If any of the five folding directions leads to a superior fitness to the original direction, this neighbor will replace the current solution.

### Termination condition

Genetic algorithm requires a termination condition to stop the evolutionary process and return the final result. In this study, the experiments ran ERS-GA and HHGA for a maximum of 200 generations. The best chromosome of the population is then returned as the final result.

## Numerical Results

Table 2 lists the eight benchmark sequences in our experiments. These sequences have been used for the 2D square HP model [20]; however, in the 2D triangular HP model the minimum energy of these benchmarks was still unknown. The comparison with previous studies provided a means of demonstrating the effectiveness of the method described here.

**Table 2:** The benchmarks for the 2D triangular lattice HP model.

Seq.	Length	Protein Sequence
1	20	(HP)^2^PH(HP)^2^(PH)^2^HP(PH)^2^

2	24	H^2^P^2^(HP^2^)^6^H^2^

3	25	P^2^HP^2^(H^2^P^4^)^3^H^2^

4	36	P(P^2^H^2^)^2^P^5^H^5^(H^2^P^2^)^2^P^2^H(HP^2^)^2^

5	48	P^2^H(P^2^H^2^)^2^P^5^H^10^P^6^(H^2^P^2^)^2^HP^2^H^5^

6	50	H^2^(PH)^3^PH^4^PH(P^3^H)^2^P^4^(HP^3^)^2^HPH^4^(PH)^3^PH^2^

7	60	P(PH^3^)^2^H^5^P^3^H^10^PHP^3^H^12^P^4^H^6^PH^2^PHP

8	64	H^12^(PH)^2^((P^2^H^2^)^2^P^2^H)^3^(PH)^2^H^11^

The experiments were conducted in two steps. First, ERS-GA was used to predict the protein structure to evaluate the efficacy of this method. Tables 3 and 4 summarize the results and compare them with prior work. According to the results in Table 3, the proposed ERS-GA significantly outperforms simple genetic algorithm (SGA) and hybrid genetic algorithm (HGA).

**Table 3:** Comparison of the proposed approach with the simple genetic algorithm (SGA) and hybrid genetic algorithm (HGA).

Seq.	Length	SGA [14]	HGA [14]	ERS-GA
1	20	-11	-**15**	**-15**

2	24	-10	-**13**	**-13**

3	25	-10	-10	**-12**

4	36	-16	-19	**-20**

5	48	-26	-**32**	**-32**

6	50	-21	-23	**-30**

7	60	-40	-46	**-55**

8	64	-33	-46	**-47**

Figures in bold indicate the lowest energy.

**Table 4:** Comparison of a hybrid of hill-climbing and GA (HHGA) with the tabu search (TS).

Seq.	Length	TS [15]	HHGA	Conformation
1	20	**-15**	**-15**	Fig. 6(a)

2	24	**-17**	**-17**	Fig. 6(b)

3	25	**-12**	**-12**	Fig. 6(c)

4	36	**-24**	-23	Fig. 6(d)

5	48	-40	**-41**	Fig. 6(e)

6	50	-	**-38**	Fig. 6(f)

7	60	**-70**	-66	Fig. 6(g)

8	64	-50	**-63**	Fig. 6(h)

Figures in bold indicate the lowest energy.

**Figure 6:**
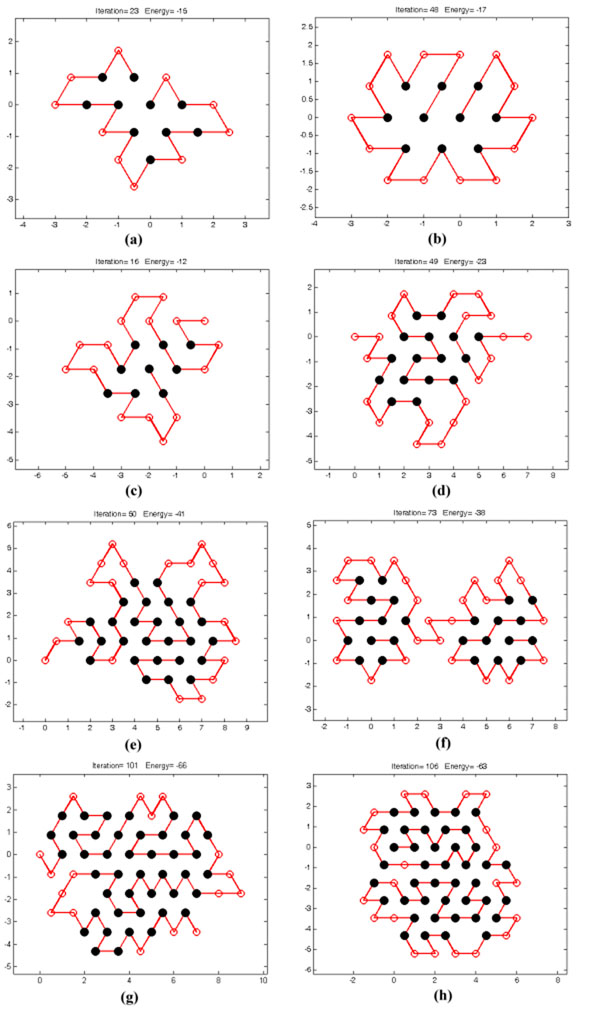
(a) to (h) Results of the structure of eight protein sequences.

Next, the HHGA integrates the hill-climbing local search into the ERS-GA approach for performance improvement. Table 5 shows that this hybrid algorithm, i.e., HHGA, can effectively enhance the performance and performs comparably with the tabu search proposed by [15]. This comparative outcome demonstrates that HHGA is a similarly good approach as the state-of-the-art method in protein structure prediction. Figure 6 plots the structures obtained from HHGA for eight protein sequences.

**Table 5:** Comparison of ERS-GA with HHGA in free energy obtained (Mean/Best) and average running time.

Seq.	Len.	Label	ERS-GA	HHGA	Conformation
1	20	Mean/Best	-12.5/-15	**-14.73/-15**	Fig. 6(a)
			
		Avg. Run Time	24.24	273.23	

2	24	Mean/Best	-10.2/-13	**-14.93/-17**	Fig. 6(b)
			
		Avg. Run Time	65.78	378.99	

3	25	Mean/Best	-8.47/-12	**-11.57/-12**	Fig. 6(c)
			
		Avg. Run Time	70.52	403.84	

4	36	Mean/Best	-16.17/-20	**-21.27/-23**	Fig. 6(d)
			
		Avg. Run Time	135.68	713.55	

5	48	Mean/Best	-28.13/-32	**-37.3/-41**	Fig. 6(e)
			
		Avg. Run Time	246.71	1173.2	

6	50	Mean/Best	-25.3/-30	**34.1/-38**	Fig. 6(f)
			
		Avg. Run Time	254.67	1246.1	

7	60	Mean/Best	-49.43/-55	**-61.83/-66**	Fig. 6(g)
			
		Avg. Run Time	366.38	1878.3	

8	64	Mean/Best	-42.37/-47	**-56.53/-63**	Fig. 6(h)
			
		Avg. Run Time	423.13	1944.7	

Figures in bold indicate the lowest energy.

Table 5 further presents the comparison of the ERS-GA with the HHGA, where each algorithm was run for 30 times. The average running time was measured on Intel i7-920 machines. The experimental results show that HHGA achieves better solution quality, i.e. lower energy, than ERS-GA does on all the benchmarks. This validates the effectiveness of the local search in HHGA. On the other hand, HHGA gains this advantage at the cost of running time.

## Conclusions

In the *ab initio* technique, the lattice model is one of the most frequently used methods in protein structure prediction. From visual comparison, however, it was found that the 2D triangular lattice model can yield better structure modeling sequences and prediction for proteins with short primary amino acid sequences. Meanwhile, it was realized that the 2D triangular lattice model has rarely been used in protein structure prediction.

This paper has highlighted this interesting issue and provides a short introduction to the working method for 2D triangular lattice models. Furthermore, the paper proposes the genetic algorithm with elite-based reproduction strategy (ERS-GA) and a hybrid of hill-climbing and genetic algorithms (HHGA) for protein structure prediction on the 2D triangular lattice. The simulation results show that ERS-GA and HHGA can successfully be applied to the problem of protein structure prediction. The satisfactory simulation results validate the effectiveness of the proposed algorithms; in addition, they demonstrate that the 2D triangular lattice model is promising for protein structure prediction.

## Competing interests

The authors declare that they have no competing interests.

## Authors' contributions

SSC carried out studies on the protein folding prediction models, participated in the design and experiments of the genetic algorithm, and drafted the manuscript. LCJ conceived of the study and participated in the design of genetic algorithm. TCK conceived of the study, participated in the design and experiments of the genetic algorithm, and drafted the manuscript.

All authors read and approved the final manuscript.
